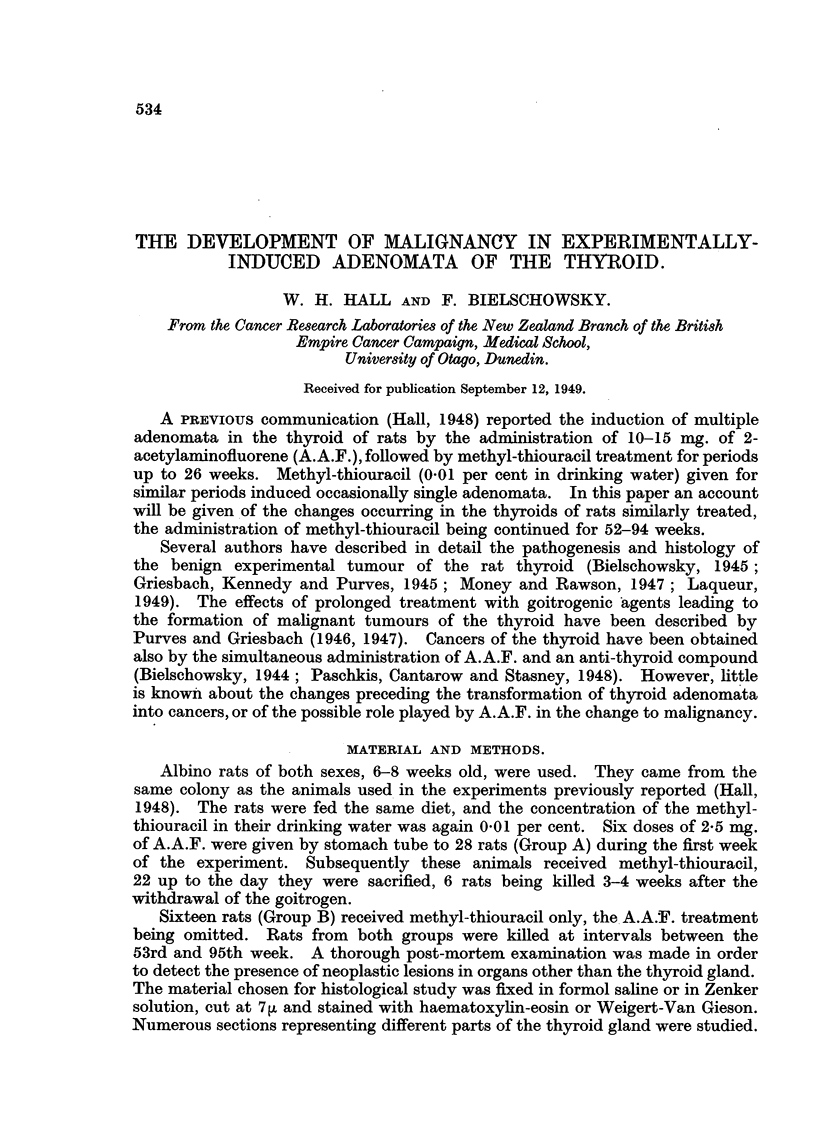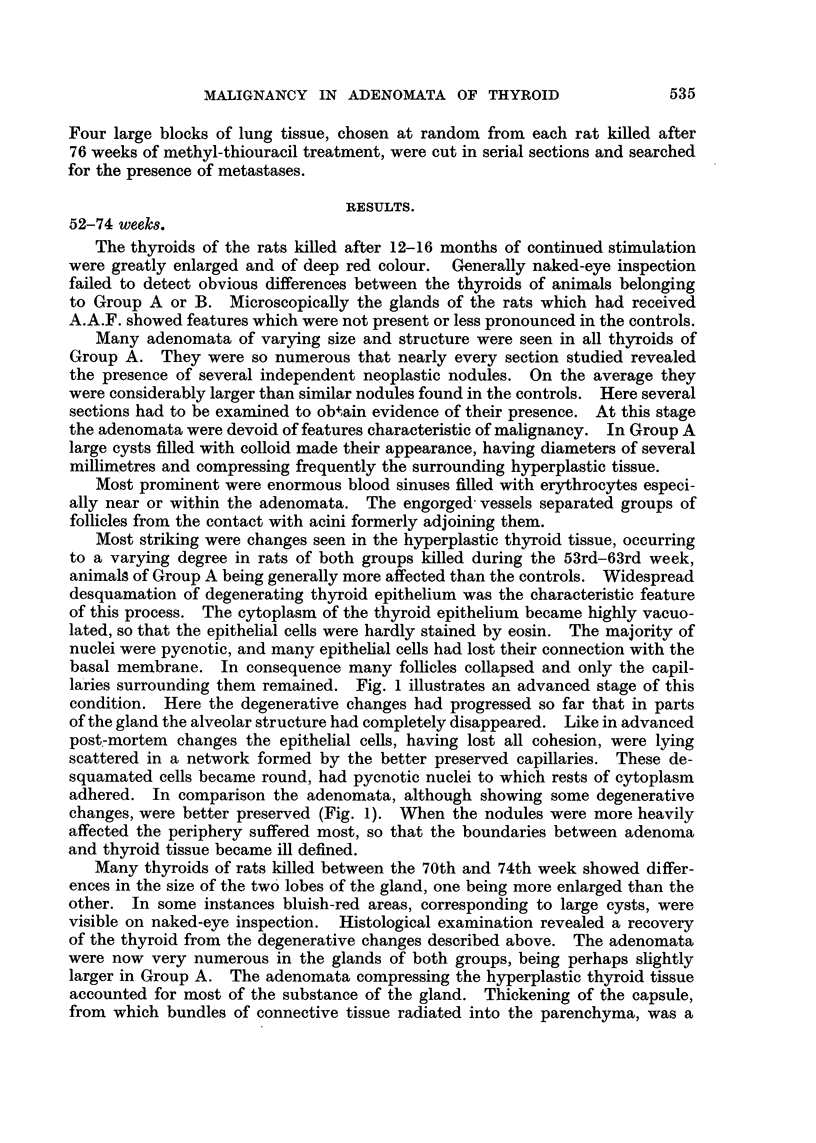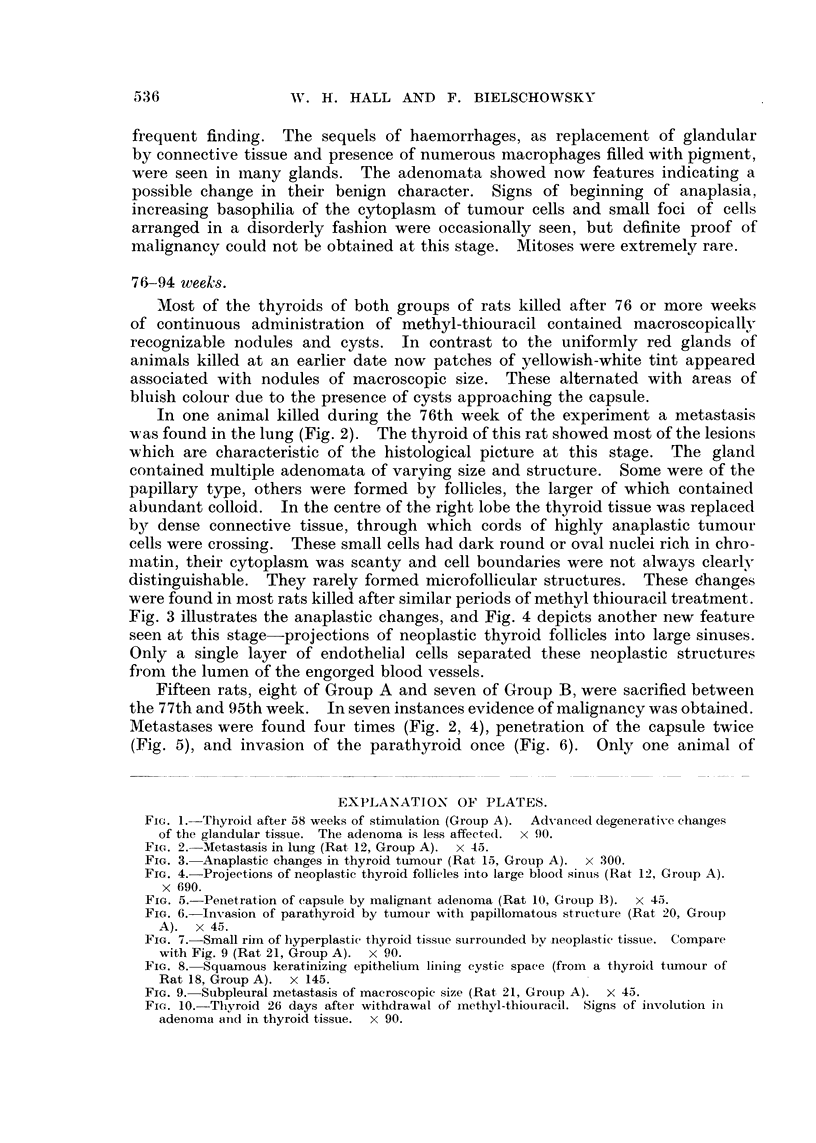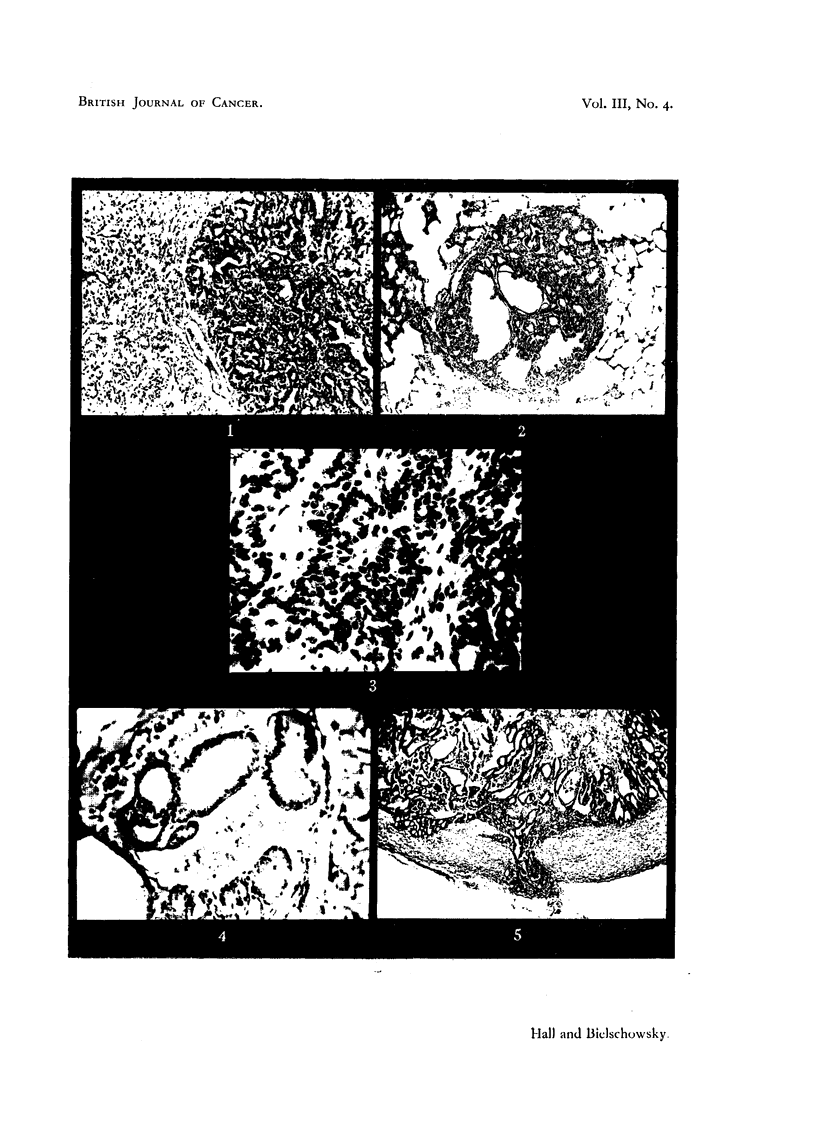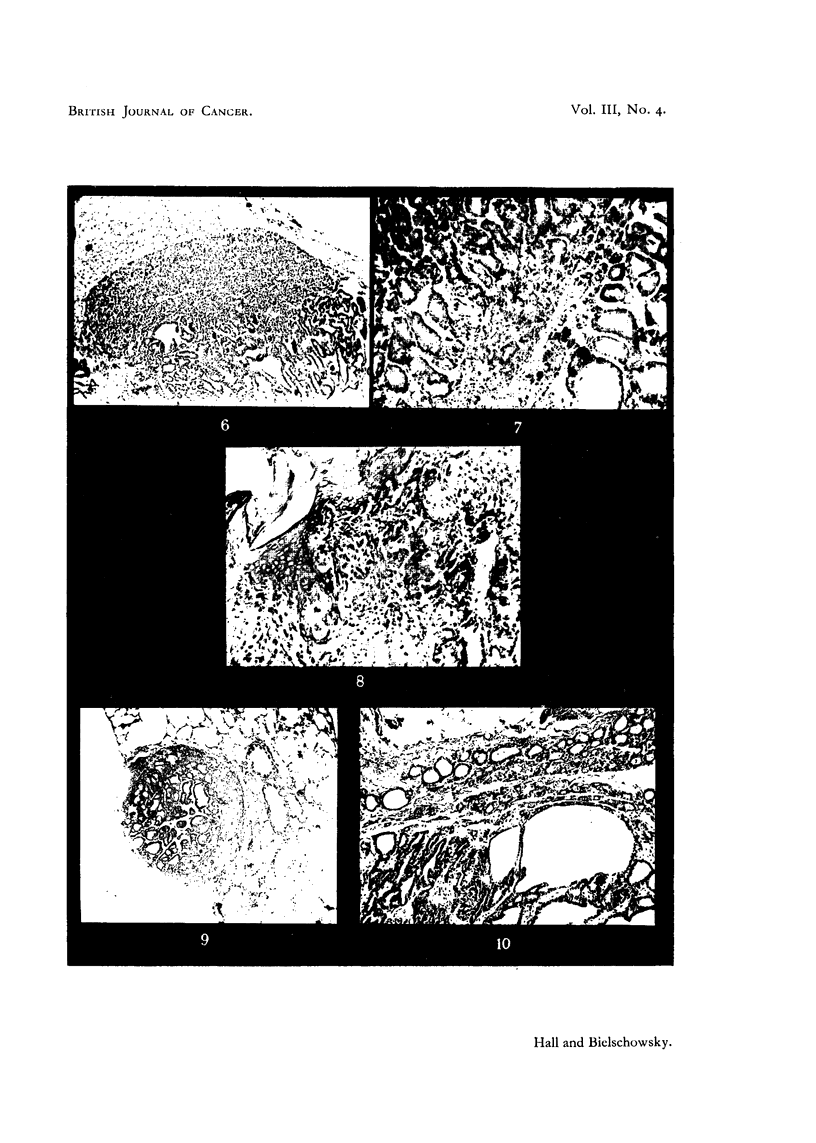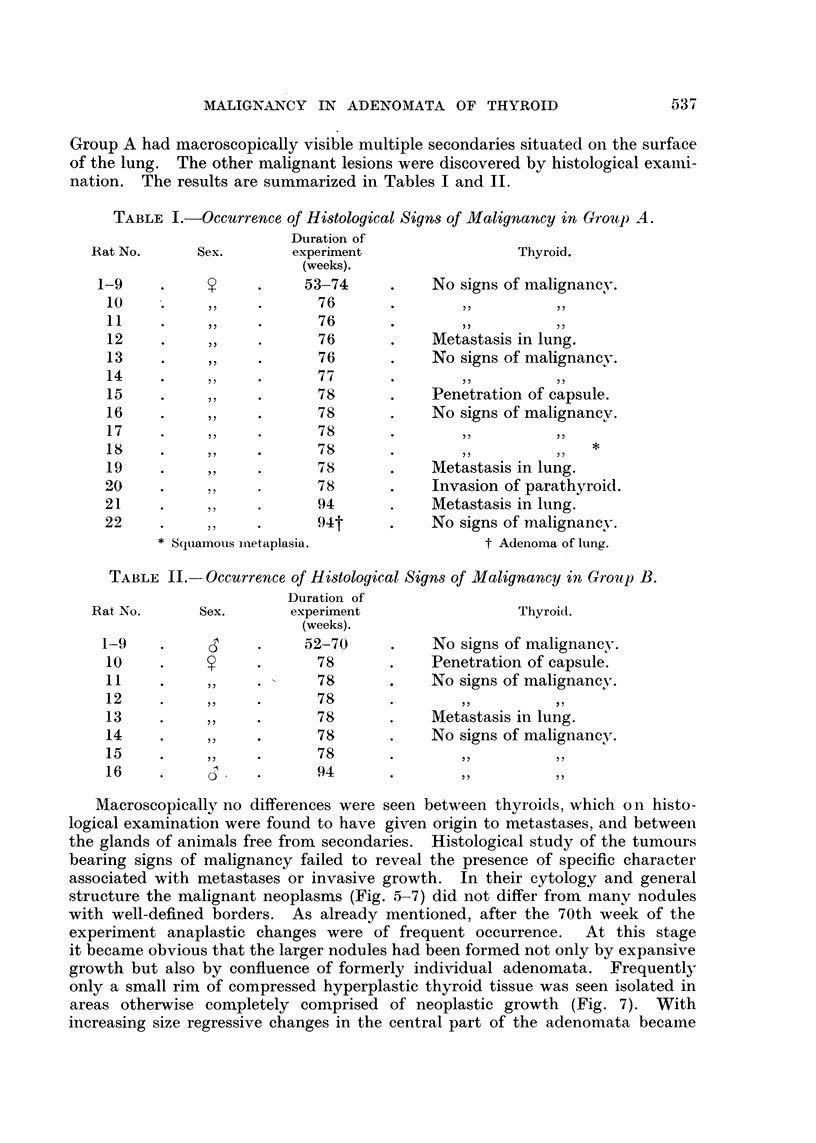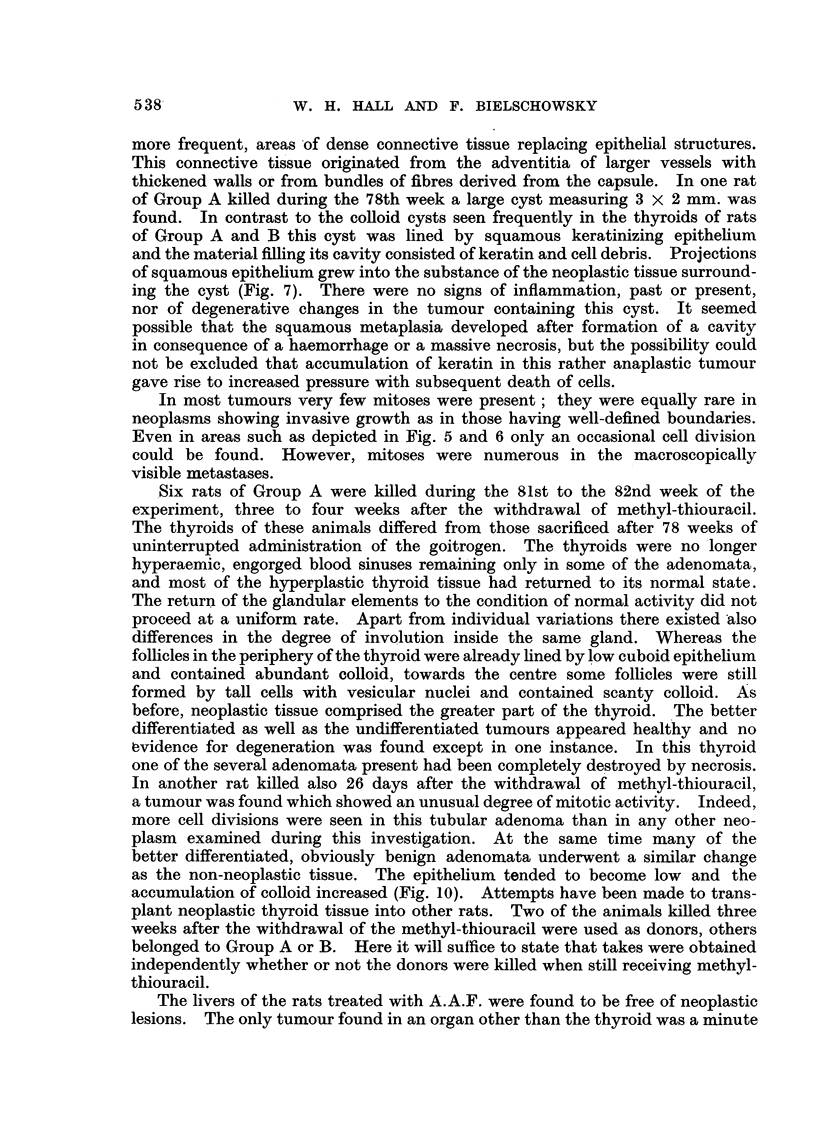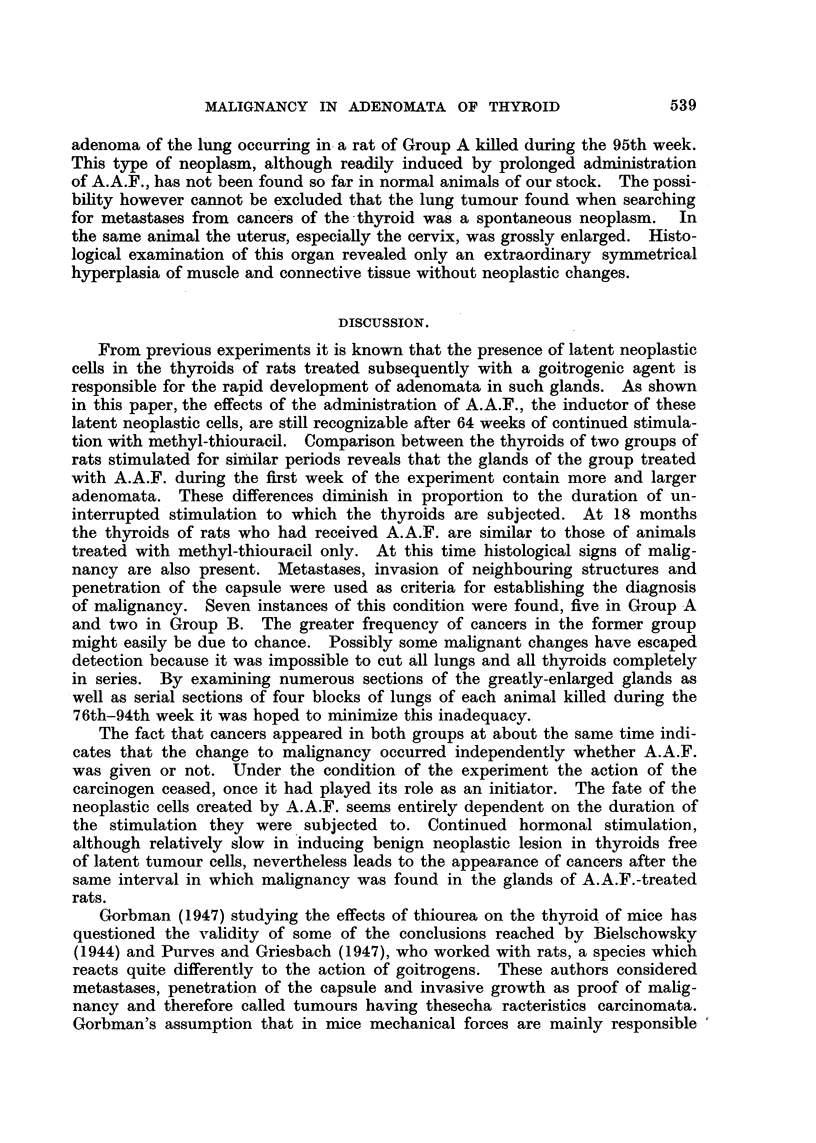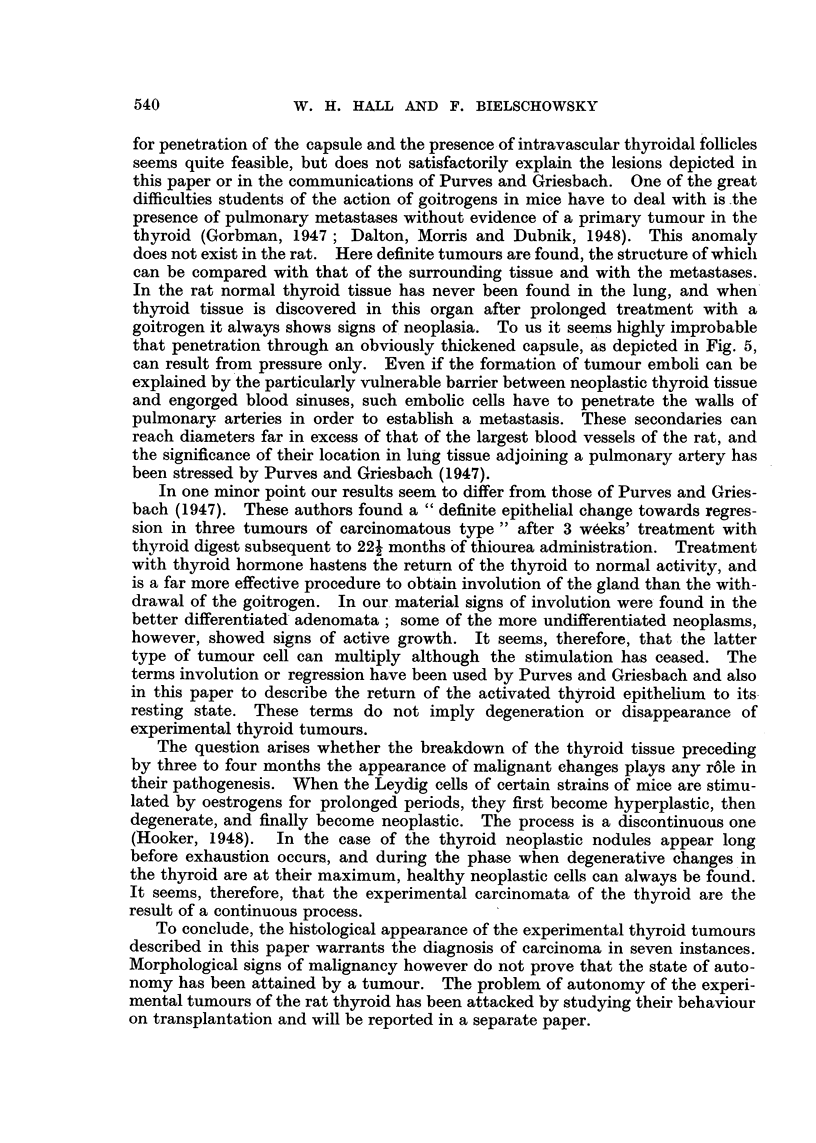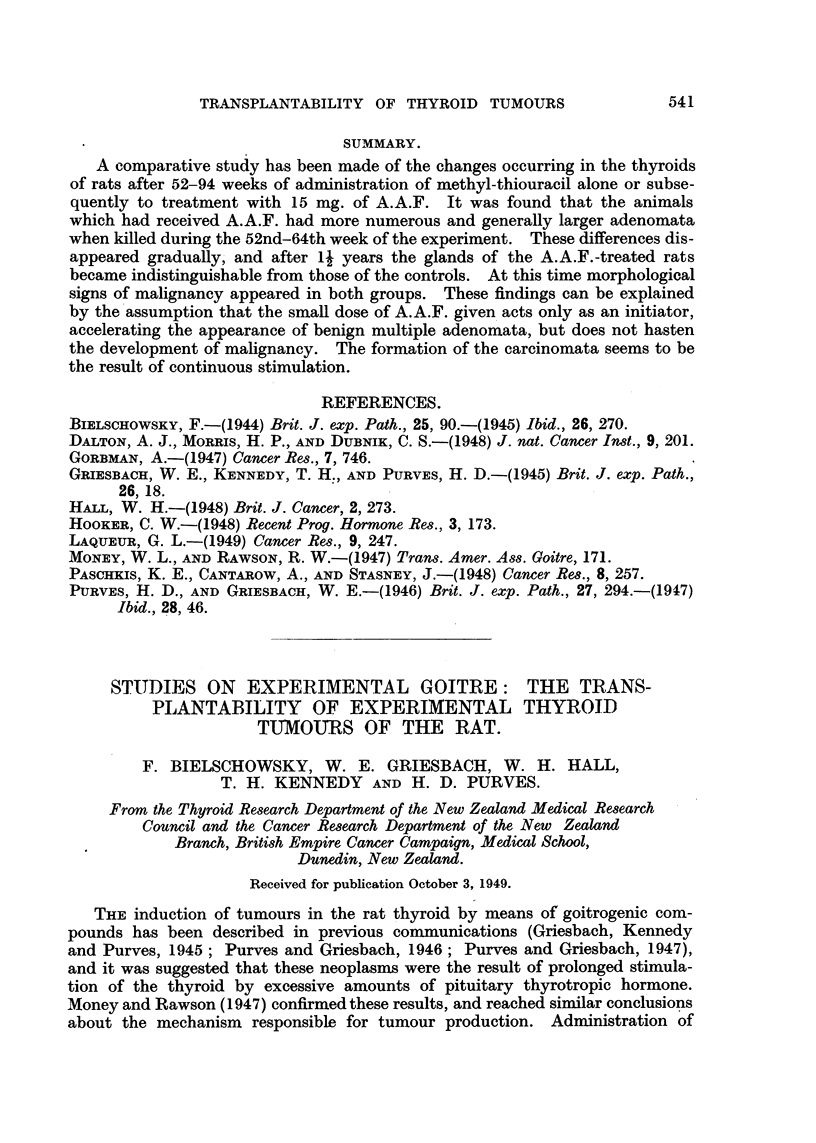# The Development of Malignancy in Experimentally-Induced Adenomata of the Thyroid

**DOI:** 10.1038/bjc.1949.57

**Published:** 1949-12

**Authors:** W. H. Hall, F. Bielschowsky

## Abstract

**Images:**


### THE DEVELOPMENT OF MALIGNANCY IN EXPERIMENTALLY-INDUCED ADENOMATA OF THE THYROID.

W. H. HALL AND F. BIELSCHOWSKY.

*From the Cancer Research Laboratories of the New Zealand Branch of the British Empire Cancer Campaign, Medical School, University of Otago, Dunedin.*

Received for publication September 12, 1949.

A PREVIOUS communication (Hall, 1948) reported the induction of multiple adenomata in the thyroid of rats by the administration of 10–15 mg. of 2-acetylaminofluorene (A.A.F.), followed by methyl-thiouracil treatment for periods up to 26 weeks. Methyl-thiouracil (0·01 per cent in drinking water) given for similar periods induced occasionally single adenomata. In this paper an account will be given of the changes occurring in the thyroids of rats similarly treated, the administration of methyl-thiouracil being continued for 52–94 weeks.

Several authors have described in detail the pathogenesis and histology of the benign experimental tumour of the rat thyroid (Bielschowsky, 1945; Griesbach, Kennedy and Purves, 1945; Money and Rawson, 1947; Laqueur, 1949). The effects of prolonged treatment with goitrogenic agents leading to the formation of malignant tumours of the thyroid have been described by Purves and Griesbach (1946, 1947). Cancers of the thyroid have been obtained also by the simultaneous administration of A.A.F. and an anti-thyroid compound (Bielschowsky, 1944; Paschkis, Cantarow and Stasney, 1948). However, little is known about the changes preceding the transformation of thyroid adenomata into cancers, or of the possible role played by A.A.F. in the change to malignancy.

### MATERIAL AND METHODS.

Albino rats of both sexes, 6–8 weeks old, were used. They came from the same colony as the animals used in the experiments previously reported (Hall, 1948). The rats were fed the same diet, and the concentration of the methyl-thiouracil in their drinking water was again 0·01 per cent. Six doses of 2·5 mg. of A.A.F. were given by stomach tube to 28 rats (Group A) during the first week of the experiment. Subsequently these animals received methyl-thiouracil, 22 up to the day they were sacrified, 6 rats being killed 3–4 weeks after the withdrawal of the goitrogen.

Sixteen rats (Group B) received methyl-thiouracil only, the A.A.F. treatment being omitted. Rats from both groups were killed at intervals between the 53rd and 95th week. A thorough post-mortem examination was made in order to detect the presence of neoplastic lesions in organs other than the thyroid gland. The material chosen for histological study was fixed in formol saline or in Zenker solution, cut at 7$\mu$ and stained with haematoxylin-eosin or Weigert-Van Gieson. Numerous sections representing different parts of the thyroid gland were studied.

Four large blocks of lung tissue, chosen at random from each rat killed after 76 weeks of methyl-thiouracil treatment, were cut in serial sections and searched for the presence of metastases.

### RESULTS.

*52–74 weeks.*

The thyroids of the rats killed after 12–16 months of continued stimulation were greatly enlarged and of deep red colour. Generally naked-eye inspection failed to detect obvious differences between the thyroids of animals belonging to Group A or B. Microscopically the glands of the rats which had received A.A.F. showed features which were not present or less pronounced in the controls.

Many adenomata of varying size and structure were seen in all thyroids of Group A. They were so numerous that nearly every section studied revealed the presence of several independent neoplastic nodules. On the average they were considerably larger than similar nodules found in the controls. Here several sections had to be examined to obtain evidence of their presence. At this stage the adenomata were devoid of features characteristic of malignancy. In Group A large cysts filled with colloid made their appearance, having diameters of several millimetres and compressing frequently the surrounding hyperplastic tissue.

Most prominent were enormous blood sinuses filled with erythrocytes especially near or within the adenomata. The engorged vessels separated groups of follicles from the contact with acini formerly adjoining them.

Most striking were changes seen in the hyperplastic thyroid tissue, occurring to a varying degree in rats of both groups killed during the 53rd–63rd week, animals of Group A being generally more affected than the controls. Widespread desquamation of degenerating thyroid epithelium was the characteristic feature of this process. The cytoplasm of the thyroid epithelium became highly vacuolated, so that the epithelial cells were hardly stained by eosin. The majority of nuclei were pycnotic, and many epithelial cells had lost their connection with the basal membrane. In consequence many follicles collapsed and only the capillaries surrounding them remained. Fig. 1 illustrates an advanced stage of this condition. Here the degenerative changes had progressed so far that in parts of the gland the alveolar structure had completely disappeared. Like in advanced post-mortem changes the epithelial cells, having lost all cohesion, were lying scattered in a network formed by the better preserved capillaries. These desquamated cells became round, had pycnotic nuclei to which rests of cytoplasm adhered. In comparison the adenomata, although showing some degenerative changes, were better preserved (Fig. 1). When the nodules were more heavily affected the periphery suffered most, so that the boundaries between adenoma and thyroid tissue became ill defined.

Many thyroids of rats killed between the 70th and 74th week showed differences in the size of the two lobes of the gland, one being more enlarged than the other. In some instances bluish-red areas, corresponding to large cysts, were visible on naked-eye inspection. Histological examination revealed a recovery of the thyroid from the degenerative changes described above. The adenomata were now very numerous in the glands of both groups, being perhaps slightly larger in Group A. The adenomata compressing the hyperplastic thyroid tissue accounted for most of the substance of the gland. Thickening of the capsule, from which bundles of connective tissue radiated into the parenchyma, was a frequent finding. The sequels of haemorrhages, as replacement of glandular by connective tissue and presence of numerous macrophages filled with pigment, were seen in many glands. The adenomata showed now features indicating a possible change in their benign character. Signs of beginning of anaplasia, increasing basophilia of the cytoplasm of tumour cells and small foci of cells arranged in a disorderly fashion were occasionally seen, but definite proof of malignancy could not be obtained at this stage. Mitoses were extremely rare.

76–94 *weeks.*

Most of the thyroids of both groups of rats killed after 76 or more weeks of continuous administration of methyl-thiouracil contained macroscopically recognizable nodules and cysts. In contrast to the uniformly red glands of animals killed at an earlier date now patches of yellowish-white tint appeared associated with nodules of macroscopic size. These alternated with areas of bluish colour due to the presence of cysts approaching the capsule.

In one animal killed during the 76th week of the experiment a metastasis was found in the lung (Fig. 2). The thyroid of this rat showed most of the lesions which are characteristic of the histological picture at this stage. The gland contained multiple adenomata of varying size and structure. Some were of the papillary type, others were formed by follicles, the larger of which contained abundant colloid. In the centre of the right lobe the thyroid tissue was replaced by dense connective tissue, through which cords of highly anaplastic tumour cells were crossing. These small cells had dark round or oval nuclei rich in chromatin, their cytoplasm was scanty and cell boundaries were not always clearly distinguishable. They rarely formed microfollicular structures. These changes were found in most rats killed after similar periods of methyl thiouracil treatment. Fig. 3 illustrates the anaplastic changes, and Fig. 4 depicts another new feature seen at this stage—projections of neoplastic thyroid follicles into large sinuses. Only a single layer of endothelial cells separated these neoplastic structures from the lumen of the engorged blood vessels.

Fifteen rats, eight of Group A and seven of Group B, were sacrified between the 77th and 95th week. In seven instances evidence of malignancy was obtained. Metastases were found four times (Fig. 2, 4), penetration of the capsule twice (Fig. 5), and invasion of the parathyroid once (Fig. 6). Only one animal of

---

EXPLANATION OF PLATES.

FIG. 1.—Thyroid after 58 weeks of stimulation (Group A). Advanced degenerative changes of the glandular tissue. The adenoma is less affected. × 90.
FIG. 2.—Metastasis in lung (Rat 12, Group A). × 45.
FIG. 3.—Anaplastic changes in thyroid tumour (Rat 15, Group A). × 300.
FIG. 4.—Projections of neoplastic thyroid follicles into large blood sinus (Rat 12, Group A). × 690.
FIG. 5.—Penetration of capsule by malignant adenoma (Rat 10, Group B). × 45.
FIG. 6.—Invasion of parathyroid by tumour with papillomatous structure (Rat 20, Group A). × 45.
FIG. 7.—Small rim of hyperplastic thyroid tissue surrounded by neoplastic tissue. Compare with Fig. 9 (Rat 21, Group A). × 90.
FIG. 8.—Squamous keratinizing epithelium lining cystic space (from a thyroid tumour of Rat 18, Group A). × 145.
FIG. 9.—Subpleural metastasis of macroscopic size (Rat 21, Group A). × 45.
FIG. 10.—Thyroid 26 days after withdrawal of methyl-thiouracil. Signs of involution in adenoma and in thyroid tissue. × 90.

1

2

3

4

5

Hall and Bielschowsky.

6

7

8

9

10

Hall and Bielschowsky.

Group A had macroscopically visible multiple secondaries situated on the surface of the lung. The other malignant lesions were discovered by histological examination. The results are summarized in Tables I and II.

TABLE I.—*Occurrence of Histological Signs of Malignancy in Group A.*

| Rat No. | Sex. | Duration of experiment (weeks). | Thyroid. |
|---|---|---|---|
| 1–9 | ♀ | 53–74 | No signs of malignancy. |
| 10 | ,, | 76 | ,, ,, |
| 11 | ,, | 76 | ,, ,, |
| 12 | ,, | 76 | Metastasis in lung. |
| 13 | ,, | 76 | No signs of malignancy. |
| 14 | ,, | 77 | ,, ,, |
| 15 | ,, | 78 | Penetration of capsule. |
| 16 | ,, | 78 | No signs of malignancy. |
| 17 | ,, | 78 | ,, ,, |
| 18 | ,, | 78 | ,, ,, * |
| 19 | ,, | 78 | Metastasis in lung. |
| 20 | ,, | 78 | Invasion of parathyroid. |
| 21 | ,, | 94 | Metastasis in lung. |
| 22 | ,, | 94† | No signs of malignancy. |

* Squamous metaplasia. † Adenoma of lung.

TABLE II.—*Occurrence of Histological Signs of Malignancy in Group B.*

| Rat No. | Sex. | Duration of experiment (weeks). | Thyroid. |
|---|---|---|---|
| 1–9 | ♂ | 52–70 | No signs of malignancy. |
| 10 | ♀ | 78 | Penetration of capsule. |
| 11 | ,, | 78 | No signs of malignancy. |
| 12 | ,, | 78 | ,, ,, |
| 13 | ,, | 78 | Metastasis in lung. |
| 14 | ,, | 78 | No signs of malignancy. |
| 15 | ,, | 78 | ,, ,, |
| 16 | ♂ | 94 | ,, ,, |

Macroscopically no differences were seen between thyroids, which on histological examination were found to have given origin to metastases, and between the glands of animals free from secondaries. Histological study of the tumours bearing signs of malignancy failed to reveal the presence of specific character associated with metastases or invasive growth. In their cytology and general structure the malignant neoplasms (Fig. 5–7) did not differ from many nodules with well-defined borders. As already mentioned, after the 70th week of the experiment anaplastic changes were of frequent occurrence. At this stage it became obvious that the larger nodules had been formed not only by expansive growth but also by confluence of formerly individual adenomata. Frequently only a small rim of compressed hyperplastic thyroid tissue was seen isolated in areas otherwise completely comprised of neoplastic growth (Fig. 7). With increasing size regressive changes in the central part of the adenomata became more frequent, areas of dense connective tissue replacing epithelial structures. This connective tissue originated from the adventitia of larger vessels with thickened walls or from bundles of fibres derived from the capsule. In one rat of Group A killed during the 78th week a large cyst measuring 3 $\times$ 2 mm. was found. In contrast to the colloid cysts seen frequently in the thyroids of rats of Group A and B this cyst was lined by squamous keratinizing epithelium and the material filling its cavity consisted of keratin and cell debris. Projections of squamous epithelium grew into the substance of the neoplastic tissue surrounding the cyst (Fig. 7). There were no signs of inflammation, past or present, nor of degenerative changes in the tumour containing this cyst. It seemed possible that the squamous metaplasia developed after formation of a cavity in consequence of a haemorrhage or a massive necrosis, but the possibility could not be excluded that accumulation of keratin in this rather anaplastic tumour gave rise to increased pressure with subsequent death of cells.

In most tumours very few mitoses were present ; they were equally rare in neoplasms showing invasive growth as in those having well-defined boundaries. Even in areas such as depicted in Fig. 5 and 6 only an occasional cell division could be found. However, mitoses were numerous in the macroscopically visible metastases.

Six rats of Group A were killed during the 81st to the 82nd week of the experiment, three to four weeks after the withdrawal of methyl-thiouracil. The thyroids of these animals differed from those sacrificed after 78 weeks of uninterrupted administration of the goitrogen. The thyroids were no longer hyperaemic, engorged blood sinuses remaining only in some of the adenomata, and most of the hyperplastic thyroid tissue had returned to its normal state. The return of the glandular elements to the condition of normal activity did not proceed at a uniform rate. Apart from individual variations there existed also differences in the degree of involution inside the same gland. Whereas the follicles in the periphery of the thyroid were already lined by low cuboid epithelium and contained abundant colloid, towards the centre some follicles were still formed by tall cells with vesicular nuclei and contained scanty colloid. As before, neoplastic tissue comprised the greater part of the thyroid. The better differentiated as well as the undifferentiated tumours appeared healthy and no evidence for degeneration was found except in one instance. In this thyroid one of the several adenomata present had been completely destroyed by necrosis. In another rat killed also 26 days after the withdrawal of methyl-thiouracil, a tumour was found which showed an unusual degree of mitotic activity. Indeed, more cell divisions were seen in this tubular adenoma than in any other neoplasm examined during this investigation. At the same time many of the better differentiated, obviously benign adenomata underwent a similar change as the non-neoplastic tissue. The epithelium tended to become low and the accumulation of colloid increased (Fig. 10). Attempts have been made to transplant neoplastic thyroid tissue into other rats. Two of the animals killed three weeks after the withdrawal of the methyl-thiouracil were used as donors, others belonged to Group A or B. Here it will suffice to state that takes were obtained independently whether or not the donors were killed when still receiving methyl-thiouracil.

The livers of the rats treated with A.A.F. were found to be free of neoplastic lesions. The only tumour found in an organ other than the thyroid was a minute adenoma of the lung occurring in a rat of Group A killed during the 95th week. This type of neoplasm, although readily induced by prolonged administration of A.A.F., has not been found so far in normal animals of our stock. The possibility however cannot be excluded that the lung tumour found when searching for metastases from cancers of the thyroid was a spontaneous neoplasm. In the same animal the uterus, especially the cervix, was grossly enlarged. Histological examination of this organ revealed only an extraordinary symmetrical hyperplasia of muscle and connective tissue without neoplastic changes.

### DISCUSSION.

From previous experiments it is known that the presence of latent neoplastic cells in the thyroids of rats treated subsequently with a goitrogenic agent is responsible for the rapid development of adenomata in such glands. As shown in this paper, the effects of the administration of A.A.F., the inductor of these latent neoplastic cells, are still recognizable after 64 weeks of continued stimulation with methyl-thiouracil. Comparison between the thyroids of two groups of rats stimulated for similar periods reveals that the glands of the group treated with A.A.F. during the first week of the experiment contain more and larger adenomata. These differences diminish in proportion to the duration of uninterrupted stimulation to which the thyroids are subjected. At 18 months the thyroids of rats who had received A.A.F. are similar to those of animals treated with methyl-thiouracil only. At this time histological signs of malignancy are also present. Metastases, invasion of neighbouring structures and penetration of the capsule were used as criteria for establishing the diagnosis of malignancy. Seven instances of this condition were found, five in Group A and two in Group B. The greater frequency of cancers in the former group might easily be due to chance. Possibly some malignant changes have escaped detection because it was impossible to cut all lungs and all thyroids completely in series. By examining numerous sections of the greatly-enlarged glands as well as serial sections of four blocks of lungs of each animal killed during the 76th–94th week it was hoped to minimize this inadequacy.

The fact that cancers appeared in both groups at about the same time indicates that the change to malignancy occurred independently whether A.A.F. was given or not. Under the condition of the experiment the action of the carcinogen ceased, once it had played its role as an initiator. The fate of the neoplastic cells created by A.A.F. seems entirely dependent on the duration of the stimulation they were subjected to. Continued hormonal stimulation, although relatively slow in inducing benign neoplastic lesion in thyroids free of latent tumour cells, nevertheless leads to the appearance of cancers after the same interval in which malignancy was found in the glands of A.A.F.-treated rats.

Gorbman (1947) studying the effects of thiourea on the thyroid of mice has questioned the validity of some of the conclusions reached by Bielschowsky (1944) and Purves and Griesbach (1947), who worked with rats, a species which reacts quite differently to the action of goitrogens. These authors considered metastases, penetration of the capsule and invasive growth as proof of malignancy and therefore called tumours having thesecha racteristics carcinomata. Gorbman's assumption that in mice mechanical forces are mainly responsible for penetration of the capsule and the presence of intravascular thyroidal follicles seems quite feasible, but does not satisfactorily explain the lesions depicted in this paper or in the communications of Purves and Griesbach. One of the great difficulties students of the action of goitrogens in mice have to deal with is the presence of pulmonary metastases without evidence of a primary tumour in the thyroid (Gorbman, 1947 ; Dalton, Morris and Dubnik, 1948). This anomaly does not exist in the rat. Here definite tumours are found, the structure of which can be compared with that of the surrounding tissue and with the metastases. In the rat normal thyroid tissue has never been found in the lung, and when thyroid tissue is discovered in this organ after prolonged treatment with a goitrogen it always shows signs of neoplasia. To us it seems highly improbable that penetration through an obviously thickened capsule, as depicted in Fig. 5, can result from pressure only. Even if the formation of tumour emboli can be explained by the particularly vulnerable barrier between neoplastic thyroid tissue and engorged blood sinuses, such embolic cells have to penetrate the walls of pulmonary arteries in order to establish a metastasis. These secondaries can reach diameters far in excess of that of the largest blood vessels of the rat, and the significance of their location in lung tissue adjoining a pulmonary artery has been stressed by Purves and Griesbach (1947).

In one minor point our results seem to differ from those of Purves and Griesbach (1947). These authors found a " definite epithelial change towards regression in three tumours of carcinomatous type " after 3 weeks' treatment with thyroid digest subsequent to 22½ months of thiourea administration. Treatment with thyroid hormone hastens the return of the thyroid to normal activity, and is a far more effective procedure to obtain involution of the gland than the withdrawal of the goitrogen. In our material signs of involution were found in the better differentiated adenomata ; some of the more undifferentiated neoplasms, however, showed signs of active growth. It seems, therefore, that the latter type of tumour cell can multiply although the stimulation has ceased. The terms involution or regression have been used by Purves and Griesbach and also in this paper to describe the return of the activated thyroid epithelium to its resting state. These terms do not imply degeneration or disappearance of experimental thyroid tumours.

The question arises whether the breakdown of the thyroid tissue preceding by three to four months the appearance of malignant changes plays any rôle in their pathogenesis. When the Leydig cells of certain strains of mice are stimulated by oestrogens for prolonged periods, they first become hyperplastic, then degenerate, and finally become neoplastic. The process is a discontinuous one (Hooker, 1948). In the case of the thyroid neoplastic nodules appear long before exhaustion occurs, and during the phase when degenerative changes in the thyroid are at their maximum, healthy neoplastic cells can always be found. It seems, therefore, that the experimental carcinomata of the thyroid are the result of a continuous process.

To conclude, the histological appearance of the experimental thyroid tumours described in this paper warrants the diagnosis of carcinoma in seven instances. Morphological signs of malignancy however do not prove that the state of autonomy has been attained by a tumour. The problem of autonomy of the experimental tumours of the rat thyroid has been attacked by studying their behaviour on transplantation and will be reported in a separate paper.

SUMMARY.

A comparative study has been made of the changes occurring in the thyroids of rats after 52–94 weeks of administration of methyl-thiouracil alone or subsequently to treatment with 15 mg. of A.A.F. It was found that the animals which had received A.A.F. had more numerous and generally larger adenomata when killed during the 52nd–64th week of the experiment. These differences disappeared gradually, and after 1½ years the glands of the A.A.F.-treated rats became indistinguishable from those of the controls. At this time morphological signs of malignancy appeared in both groups. These findings can be explained by the assumption that the small dose of A.A.F. given acts only as an initiator, accelerating the appearance of benign multiple adenomata, but does not hasten the development of malignancy. The formation of the carcinomata seems to be the result of continuous stimulation.

### REFERENCES.

BIELSCHOWSKY, F.—(1944) *Brit. J. exp. Path.*, **25**, 90.—(1945) *Ibid.*, **26**, 270.
DALTON, A. J., MORRIS, H. P., AND DUBNIK, C. S.—(1948) *J. nat. Cancer Inst.*, **9**, 201.
GORBMAN, A.—(1947) *Cancer Res.*, **7**, 746.
GRIESBACH, W. E., KENNEDY, T. H., AND PURVES, H. D.—(1945) *Brit. J. exp. Path.*, **26**, 18.
HALL, W. H.—(1948) *Brit. J. Cancer*, **2**, 273.
HOOKER, C. W.—(1948) *Recent Prog. Hormone Res.*, **3**, 173.
LAQUEUR, G. L.—(1949) *Cancer Res.*, **9**, 247.
MONEY, W. L., AND RAWSON, R. W.—(1947) *Trans. Amer. Ass. Goitre*, 171.
PASCHKIS, K. E., CANTAROW, A., AND STASNEY, J.—(1948) *Cancer Res.*, **8**, 257.
PURVES, H. D., AND GRIESBACH, W. E.—(1946) *Brit. J. exp. Path.*, **27**, 294.—(1947) *Ibid.*, **28**, 46.